# The College Edifice, &c.

**Published:** 1857-02

**Authors:** 


### THE COLLEGE EDIFICE, &C.

We learn that it is a matter of surprise with some, that the Faculty of the Atlanta Medical College are able to erect a large and costly Edifice, and send a Professor to Europe, and to do divers other things (among which may be, perhaps, the purchase of, what is said to be, the finest Chemical Apparatus in the Southern country) that have not been thought possible, except by Institutions that have been endowed, or those of long standing.

To this very natural curiosity (while in every instance it may not proceed from the best motives, and might not, therefore, be entitled to gratification), we would reply, that there has been such entire confidence in the success of the Institution, and such unity of purpose in the Faculty, that they have not hesitated to embark their means in the enterprise, which must now be admitted upon all hands, to be one of the most successful, that has ever been undertaken in the United States; so far as we know or believe, having only been exceeded by the University of Nashville, in the number of the class in attendance, at the same period from its commencement.

As stated in our last number, we can assure those who feel interested, that the College Edifice will be in readiness for the next term; and in addition, that we shall be prepared with proper anatomical material, and all the necessary appliances for successful teaching.

The growth of Atlanta, itself, has been an enigma to many, but notwithstanding incredulity and predictions of evil, the city has continued to grow, and is now admitted to have a successful future; and with the almost certainty of another railroad, and the strong prospect of it being the future capital of the great State of Georgia, it is indeed difficult to put a limit upon the progress of the place. We can, then, point those who wonder at the remarkable success of the Medical College, to the fact, that it is in the city of Atlanta, whose history has been, and will continue to be onward, notwithstanding every effort to the contrary, upon the part of those whose interests have been, or are supposed to be, in conflict with her prosperity. But to return from this digression, and to conclude what was intended to be little more than a paragraph, we would say in conclusion, that from all the information we can get, and the data from which we have a right to judge, we shall number in the next class a large increase upon the last.

